# Multicenter cross-sectional study of HTLV-1 prevalence and associated risk factors in epidemiologically relevant groups across Brazil

**DOI:** 10.3389/fpubh.2025.1511374

**Published:** 2025-03-03

**Authors:** Carlos Brites, Prince Baffour Tonto, Antonio C. Vallinoto, Onayane dos Santos Oliveira, Simone Simionatto, Monica Bay, Tania Reuter, Monica M. Gomes-da-Silva, Melissa Medeiros, Rafaela Mayoral, Estela Luz, Michael Rocha, Hareton Vechi, Bobby Brooke Herrera

**Affiliations:** ^1^Hospital Universitário Professor Edgard Santos, Universidade Federal da Bahia, Salvador, Brazil; ^2^Department of Medicine, Division of Allergy, Immunology, and Infectious Diseases, and Child Health Institute of New Jersey, Rutgers Robert Wood Johnson Medical School, Rutgers University, New Brunswick, NJ, United States; ^3^Rutgers Global Health Institute, Rutgers University, New Brunswick, NJ, United States; ^4^Laboratório de Virologia, Instituto de Ciências Biológicas, Universidade Federal do Pará, Belém, Brazil; ^5^Laboratório de Pesquisa em Ciências da Saúde, Universidade Federal Grande Dourados, Dourados, Brazil; ^6^Departamento de Infectologia, Universidade Federal do Rio Grande do Norte, Natal, Brazil; ^7^Centro de Pesquisa Clinica em Infectologia, Hospital Universitário Cassiano Antonio de Moraes, Universidade Federal do Espirito do Espirito Santo, Vitoria, Brazil; ^8^Hospital de Clinicas, Universidade Federal do Paraná, Curitiba, PR, Brazil; ^9^Hospital São josé de Doenças Infecciosas, Fortaleza, Brazil; ^10^Centro Universitário Unichristus, Fortaleza, Ceará, Brazil; ^11^Instituto Brasileiro de Investigação do Torax, Salvador, BA, Brazil; ^12^Instituto de Medicina Tropical, Universidade Federal do Rio Grande do Norte, Natal, Brazil; ^13^Escola Multicampi de Ciências Médicas, Universidade Federal do Rio Grande do Norte, Caicó, Rio Grande do Norte, Brazil

**Keywords:** multicenter, cross-sectional study, HTLV-1 prevalence, risk factors, epidemiologically relevant groups

## Abstract

**Background:**

Human T cell lymphotropic virus type 1 (HTLV-1) is highly endemic in Brazil, necessitating surveillance studies to understand its epidemiology. While previous research has focused on either specific cities or populations, there is a need for multicenter studies encompassing epidemiologically relevant populations to ascertain more accurate prevalence rates and predictors of HTLV-1 infection in the country.

**Methods:**

We conducted a multicenter, cross-sectional study involving 3,184 participants across seven cities and five study populations in Brazil. Blood samples were collected, and the prevalence of HTLV-1 infection was determined by enzyme-linked immunosorbent assay (ELISA) and Western blot. Binary logistic regression analysis was used to determine risk factors of HTLV-1 infection.

**Results:**

Among the total study population, 1,135 (35.7%) were aged >40 years and 1,704 (53.5%) were female. The overall prevalence of HTLV-1 infection was 0.5% (95% CI: 0.3–0.8), with variation observed among the cities or study populations. Factors associated with HTLV-1 infection included age > 40 years (OR, 8.867; 95% CI: 1.824–43.099), female gender (OR, 4.604; 95% CI: 1.184–17.903), and Hepatitis C virus (HCV) infection (OR, 13.995; 95% CI: 2.374–82.506). The identification of older age and female gender, coupled with the high prevalence of HTLV-1 in HIV-positive patients, suggests sexual transmission as the primary route of HTLV-1 infection.

**Conclusion:**

Our study reveals varied prevalence rates of HTLV-1 infection across diverse populations and cities in Brazil. The association of older age, female gender, and HCV, emphasizes the need for tailored interventions to prevent HTLV-1 transmission.

## Introduction

Surveillance of infectious pathogens is fundamental to public health, enabling the development and implementation of strategies to curb their spread and alleviate disease burden. Effective surveillance requires diverse and robust testing methodologies capable of identifying pathogens, particularly those with the ability to establish latent infections within host cells and transmit infections inapparently (1). Among these pathogens, Human T cell lymphotropic virus type 1 (HTLV-1) emerges as a significant concern.

HTLV-1, the first human oncogenic retrovirus discovered (2), presents a multifaceted challenge to public health due to its modes of transmission and associated debilitating conditions. Transmission predominantly occurs through sexual intercourse (3), vertical transmission via pregnancy or prolonged breastfeeding (4), and exposure to contaminated blood products or shared needles or syringes (5). The repercussions of HTLV-1 are severe, encompassing conditions such as Adult T-cell leukemia/lymphoma (ATLL) (6), characterized by a dismal prognosis and a median survival of less than a year, and HTLV-1-associated myelopathy/tropical spastic paraparesis (HAM/TSP) (7, 8), associated with a reduced quality of life and lifespan. HTLV-1 infection can also cause inflammatory syndromes including arthritis (9), uveitis (10), and infective dermatitis (11).

The primary cellular target of HTLV-1 is CD4+ T-cells, although it can also infect CD8+ T-cells, dendritic cells and monocytes to a lesser extent. Unlike HIV, which efficiently infects CD4+ T-cells through cell-free virions, HTLV-1 relies on host cell machinery for cell-to-cell transmission of its targets. Following viral replication in CD4+ T cells and formation of virions, HTLV-1 transfer viral particles to uninfected cells through multiple mechanisms, including the formation of a viral synapse, formation of biofilm-like structures containing virions, the establishment of cell–cell conjugates, the trafficking of extracellular vesicles bearing viral components (12).

Despite its global prevalence with an estimated 5–20 million people infected, HTLV-1 infection is endemic in diverse regions, including but not limited to Japan, the Caribbean, Sub-Saharan Africa, Central and South America, and the Middle East (13–16). There are currently no approved vaccines or anti-retroviral therapeutics to prevent or help manage HTLV-1 infection, due in large part to the lack of funding dedicated to HTLV-1 research. Treatment options are limited to supportive care, symptomatic management, and addressing complications associated with HTLV-1-related diseases, particularly ATLL and HAM/TSP (17, 18).

Within South America, Brazil is a focal point for HTLV-1 endemicity with an estimated 800,000 people infected (19). However, prevalence rates vary considerably among different populations and geographic areas within the country. Notably, higher prevalence rates are typically observed in the northern and northeastern regions of the country compared to other areas (20). Historically, individuals with underlying comorbidities such as HIV infection and tuberculosis have been shown to be highly susceptible to HTLV-1 infection in Brazil, as evidenced by higher but variable prevalence rates across different geographical locations. Notably, reported HTLV-1 prevalence rates range from 1.4 to 21.1% among HIV patients (21), and 4.3 to 10.8% among tuberculosis (TB) patients (22). Furthermore, pregnant women (PW) represent a key population in HTLV-1 epidemiology due to the potential for vertical transmission of the virus. Studies have reported varying HTLV-1 prevalence rates, ranging from 0.1 to 1.1% among PW (23).

Despite numerous studies conducted in Brazil to understand HTLV-1 prevalence rates, many have been localized to specific areas with varying methodological approaches, hindering the accurate estimation of nationwide prevalence. Therefore, studies encompassing diverse regions and populations are imperative to provide a comprehensive understanding of the magnitude and potential predictors of HTLV-1 infection in the country. The present study is a multicenter, cross-sectional evaluation of HTLV-1 prevalence and associated risk factors across seven cities in five different regions (Salvador, Belem, Curitiba, Dourados, Fortaleza, Natal, Vitoria) and five epidemiologically relevant populations (general population, individuals with HIV infection, individuals with tuberculosis, pregnant women, and individuals on pre-exposure prophylaxis [PREP]) in Brazil.

## Methods

### Study design and population

**Figure 1:**
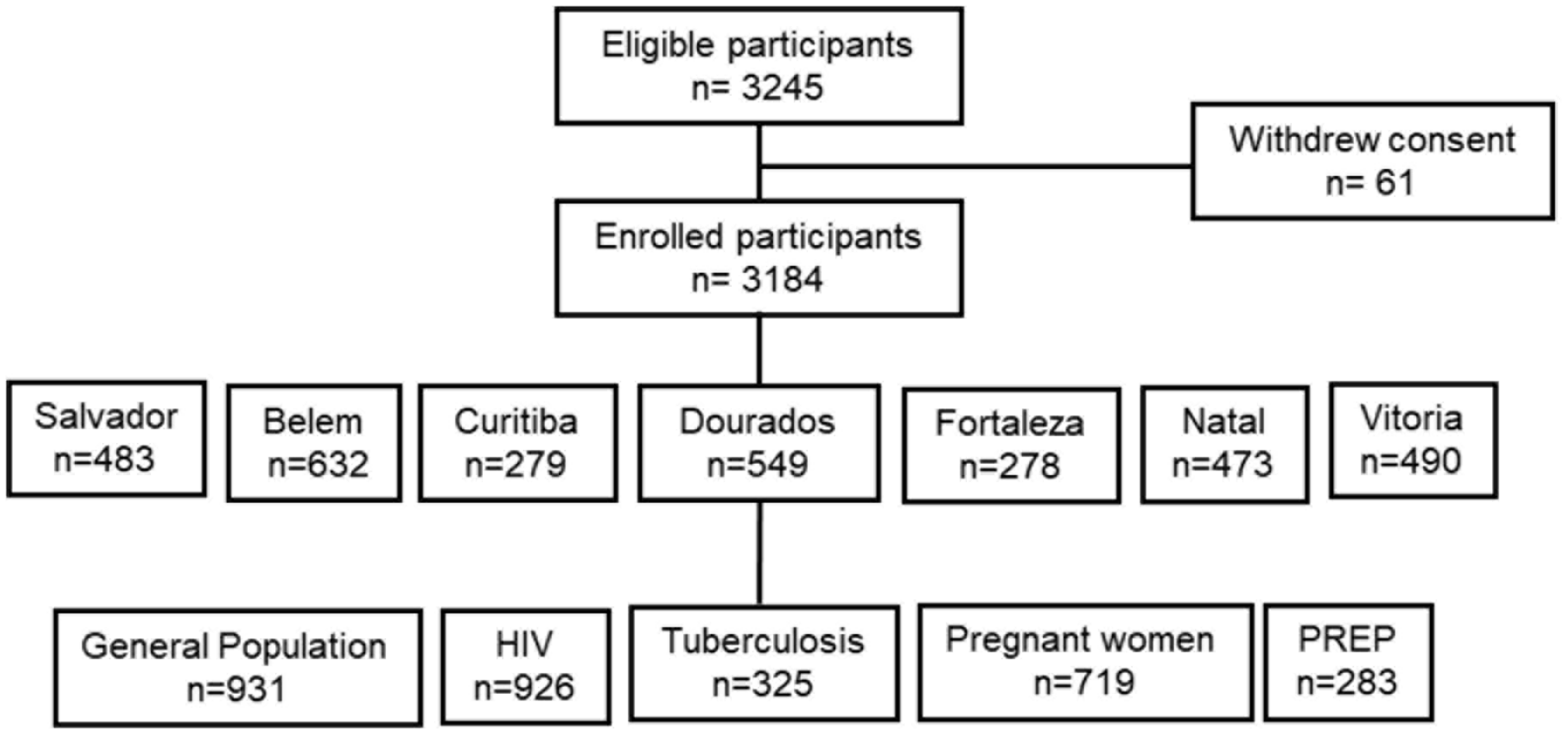
Schematic diagram showing participants in each of the selected city and study population.

Our multicenter, cross-sectional study included 3,184 participants aged from 11 to 85 years, recruited from seven distinct cities in Brazil from 2021 to 2023. These cities comprised six state capital cities: Fortaleza, Natal, and Salvador in the northeastern part of the country (capital cities of Ceará, Rio Grande do Norte, and Bahia states, respectively); Belem (capital city of Pará state); Vitória (capital of Espirito Santo state); Curitiba (capital of Paraná state); and Dourados (the second largest city of Mato Grosso do Sul state), representing the northern, southeastern, southern and central-western regions, respectively (Figure 1).

The study population comprised five groups: 926 HIV-positive patients attending HIV referral centers, 325 recently diagnosed TB patients receiving treatment at tuberculosis referral centers, 719 PW attending public antenatal care centers, and 931 patients seeking care for various other reasons at public health centers, categorized as the general population (GP) (Figure 1). An additional group of 238 participants attending an HIV prophylaxis center in Natal was included due to the lack of HTLV-1 prevalence data in this population. Furthermore, one site could only screen people with HIV due to local issues related to the COVID-19 pandemic. None of the study participants presented with symptoms of ATL or HAM/TSP, or other clinical signs associated with HTLV-1.

### Eligibility criteria

Participants were eligible for the study if they met any one of the inclusion criteria: (1) residence of the specified cities; (2) Diagnosis of HIV infection; (3) Diagnosis of tuberculosis; (4) Pregnancy; and (5) Attendance at public health centers for other medical reasons within these cities. The exclusion criterion was failure or refusal to provide informed consent.

### Background data collection

Socio-demographic and non-infectious comorbidities were solicited from the participants using a well-structured and standardized written questionnaire. The socio-demographic factors were age, gender, family income, length of education, marital status, stable relationship, ethnicity, blood transfusion, intravenous drug use, and sexual orientation. For non-infectious comorbidities, factors including diabetes mellitus, arterial hypertension, obesity and cardiovascular diseases were collected. Information on infectious comorbidities that included hepatitis B (HBV) and C (HCV) was obtained through the medical history of the participants.

### Sample size determination

The required sample size was calculated based on an estimated HTLV-1 prevalence of 0.5% among the GP and PW, 4% among HIV-positive individuals, and 10% among tuberculosis patients, with a margin of error of 0.5% for GP and PW, 1.5% for HIV and 4% for Tuberculosis. Using a 97% confidence interval, the minimum sample size was calculated to be 2,693. We used the formula: N*p (1−p)/(d^2^/Z^2^_1−*α*/2_)*(N−1) + p*(1−p) to calculate the sample size. We added an extra 10% to account for missing data and dropouts, increasing the sample size to 2,963. Additionally, 283 participants from an HIV PrEP program were included due to limited data on HTLV-1 infection within this population. 61 participants who initially satisfied the inclusion criteria, withdrew their consent, and were subsequently excluded from the study, resulting in a final sample size of 3,184 (98% response rate) (Figure 1).

### Ethics statement

Ethical clearance for demographic data and whole blood sample collection was obtained from the institutional review boards (IRB) from each clinical site, including Maternidade Climério de Oliveira, Universidade Federal da Bahia (approval number: 31650520.0.1001.5543), Universidade Federal da Grande Dourados (approval number: 31650520.0.2007.5160), Universitário Cassiano Antônio de Moraes da Universidade Federal do Espirito Santo (approval number: 31650520.0.2009.5071), Instituto de Ciências da Saúde da Universidade Federal do Para (approval number: 31650520.0.2008.0018), Hospital Universitário Onofre Lopes Da Universidade Federal do Rio Grande Do Norte (approval number: 31650520.0.2005.5292), Hospital de Clinicas da Universidade Federal do Parana (approval number: 31650520.0.2006.0096), and Hospital São Jose de Doenças Infecciosas – HSJ/Secretaria de Saúde de Fortaleza (approval number: 31650520.0.2001.5044). The study was conducted in accordance with the principles of Declaration of Helsinki. Formal written consent was obtained from all participants. This study received an exemption determination from the Rutgers Robert Wood Johnson Medical School IRB.

### Blood sampling and processing

10 mL of whole blood was collected from each participant via venipuncture into heparin-containing tubes. Blood samples were subjected to centrifugation immediately after collection for 15 min at 2000 g. Serum samples were transferred into cryotubes and stored at −20°C until further analysis. The time interval between blood collection and testing varied depending on the study site and ranged from 24 to 48 h.

### Serology and Western blotting

Detection of antibodies against HTLV was conducted by using an ELISA test kit (HTLV-1/II ELISA 4.0, MP Diagnostics, Eschwege, Germany), according to the manufacturer’s instructions. All reactive samples by ELISA were further analysed by Western blot (HTLV Blot 2.4, MP Diagnostics, Eschwege, Germany) to differentiate between exposure to HTLV-1 and HTLV-2, according to the manufacturer’s instructions.

### Statistical analysis

Categorical variables were represented as case counts and proportions, with differences between categorical variables assessed using Chi-square or Fisher’s exact tests, as appropriate. Univariate and multivariate binary logistic regression analyses were performed to assess the association between HTLV-1 prevalence and background factors. Variables used in the univariate analysis were age group, gender, stable relationship, marital status, ethnicity, sexual orientation, blood transfusion, intravenous drug use, length of education, family income, comorbidities, city, and study population. Finally, variables with *p* ≤ 0.25 in the univariate analysis were included in the multivariate logistic regression analysis. Multiple imputation (MI) method was done to replace the missing values, and the variables included in the MI model were stable relationship, blood transfusion, intravenous drug use, HCV, HBV, and sexual orientation. Statistical analyses were conducted, and plots were generated using IBM SPSS statistics software version 28 and/or GraphPad Prism 10. Finally, the map of Brazil, illustrating HTLV-1 prevalence rates, was generated using R version 4.4.1.

## Results

### Characteristics of study subjects

A total of 3,184 individuals participated in the study. Of these, 1,135 (35.7%) were aged >40 years and 1,704 (53.5%) were female (Table 1). Regarding racial characteristics, 1,699 (53.4%) subjects identified themselves as Black or mixed race. With respect to relationship, approximately 48% reported being in a stable relationship, with a majority reporting being single and never married, accounting for approximately 57% of the study cohort (Table 1). Majority of participants also self-identified as heterosexual (1,949; 61.2%), and 85 (2.7%) participants reported a prior history of blood transfusion (Table 1). Concerning educational attainment, a substantial proportion of the participants reported receiving less than 12 years of formal education (2,461; 77.3%) (Table 1). Furthermore, 2,461 (47.1%) participants reported a family income of less than 2 minimum wages.

**Table 1:** Baseline characteristics of study participants by Brazilian city.

	City							
	Salvador	Belem	Curitiba	Dourados	Fortaleza	Natal	Vitoria	Total
	*n* = 483	*n* = 632	*n* = 279	*n* = 549	*n* = 278	*n* = 473	*n* = 490	*n* = 3,184
Age groups, *n* (%)								
≤40 years	320 (66.3)	404 (63.9)	116 (41.6)	390 (71.0)	173 (62.2)	344 (72.7)	290 (59.2)	2037 (64.0)
>40 years	162 (33.5)	227 (35.9)	158 (56.6)	158 (28.8)	105 (37.8)	125 (26.4)	200 (40.8)	1,135 (35.7)
Unreported	1 (0.2)	1 (0.2)	5 (1.8)	1 (0.2)	0 (0.0)	4 (0.9)	0 (0.0)	12 (0.3)
Gender, *n* (%)								
Male	196 (40.6)	207 (32.8)	98 (35.1)	194 (35.3)	235 (84.5)	379 (80.1)	169 (34.5)	1,478 (46.4)
Female	287 (59.4)	425 (67.2)	181 (64.9)	355 (64.7)	43 (15.5)	92 (19.5)	321 (65.5)	1704 (53.5)
Unreported	0 (0.0)	0 (0.0)	0 (0.0)	0 (0.0)	0 (0.0)	2 (0.4)	0 (0.0)	2 (0.1)
Stable relationship, *n* (%)	271 (56.1)	392 (62.0)	200 (71.7)	254 (46.3)	104 (37.4)	152 (32.1)	150 (30.6)	1,523 (47.8)
Marital Status, *n* (%)								
Single	306 (63.4)	290 (45.9)	125 (44.8)	274 (49.9)	205 (73.8)	347 (73.4)	255 (52.0)	1802 (56.6)
Married	147 (30.4)	287 (45.4)	101 (36.2)	210 (38.3)	37 (13.3)	91 (19.2)	167 (34.1)	1,040 (32.7)
Divorced/Separated	14 (2.9)	30 (4.8)	35 (12.5)	43 (7.8)	14 (5.0)	24 (5.1)	47 (9.6)	207 (6.5)
Widowed	13 (2.7)	23 (3.6)	15 (5.4)	15 (2.7)	2 (0.7)	8 (1.7)	15 (3.1)	91 (2.9)
Unreported	3 (0.6)	2 (0.3)	3 (1.1)	7 (1.3)	20 (7.2)	3 (0.6)	6 (1.2)	44 (1.4)
Ethnicity, *n* (%)								
Black/Mixed	371 (76.8)	169 (26.7)	65 (23.0)	284 (52.1)	178 (64.0)	281 (59.0)	348 (71.9)	1,699 (53.4)
White	99 (20.5)	83 (13.1)	186 (67.0)	228 (41.2)	62 (22.3)	171 (36.6)	129 (25.5)	955 (30.0)
Indigenous	1 (0.2)	379 (60.0)	2 (0.7)	25 (4.5)	4 (1.5)	18 (3.8)	3 (0.6)	432 (13.6)
Asian	3 (0.6)	0 (0.0)	0 (0)	10 (1.8)	4 (1.4)	0 (0.0)	4 (0.8)	21 (0.6)
Unreported	9 (1.9)	1 (0.2)	26 (9.3)	2 (0.4)	30 (10.8)	3 (0.6)	6 (1.2)	77 (2.4)
Sexual orientation, *n* (%)								
Heterosexual	405 (83.8)	535 (84.7)	233 (83.5)	485 (88.4)	72 (25.9)	0 (0.0)	219 (44.7)	1949 (61.2)
Bisexual	21 (4.4)	27 (4.3)	6 (2.2)	21 (3.8)	17 (6.1)	58 (12.3)	5 (1.0)	155 (4.9)
Homosexual	43 (8.9)	69 (10.9)	11 (3.9)	11 (2.0)	156 (56.1)	220 (46.5)	32 (6.5)	542 (17.0)
Unreported	14 (2.9)	1 (0.1)	29 (10.4)	32 (5.8)	33 (11.9)	195 (41.2)	234 (47.8)	538 (16.9)
Blood transfusion, *n* (%)	12 (2.5)	0 (0.0)	57 (20.4)	12 (2.2)	0 (0.0)	2 (0.4)	2 (0.4)	85 (2.7)
Intravenous drug use, *n* (%)	2 (0.4)	0 (0.0)	1 (0.4)	7 (1.3)	0 (0.0)	0 (0.0)	1 (0.2)	11 (0.4)
Length of education, *n* (%)								
<12 years	256 (53.0)	618 (97.8)	260 (93.2)	408 (74.3)	134 (48.2)	436 (92.2)	349 (71.2)	2,461 (77.3)
≥12 years	221 (45.8)	13 (2.1)	1 (0.4)	134 (24.4)	117 (42.1)	34 (7.2)	131 (26.7)	651 (20.4)
Unreported	6 (1.2)	1 (0.1)	18 (6.4)	7 (1.3)	27 (9.7)	3 (0.6)	10 (2.1)	72 (2.3)
Family income, *n* (%)								
Up to 1 MW	181 (37.5)	416 (65.8)	89 (31.9)	265 (48.27)	134 (48.2)	176 (37.2)	238 (48.6)	1,499 (47.1)
2 to 5 MW	184 (38.1)	189 (29.9)	159 (57.0)	237 (43.17)	115 (41.4)	218 (46.1)	194 (39.6)	1,296 (40.7)
More than 5 MW	116 (24.0)	22 (3.5)	31 (11.1)	47 (8.6)	29 (10.4)	79 (16.7)	58 (11.8)	382 (12.0)
Unreported	2 (0.4)	5 (0.8)	0 (0.0)	0 (0.0)	0 (0.0)	0 (0.0)	0 (0.0)	7 (0.2)
Comorbidities, *n* (%)								
HCV	3 (0.62)	5 (0.8)	11 (3.9)	1 (0.2)	2 (0.7)	0 (0.0)	10 (2.0)	32 (1.0)
HBV	5 (1.0)	12 (1.9)	14 (5.0)	1 (0.2)	1 (0.4)	2 (0.4)	30 (6.1)	65 (2.0)
Diabetes mellitus	43 (8.9)	58 (9.2)	39 (13.9)	53 (9.7)	16 (5.8)	20 (4.2)	39 (8.0)	268 (8.4)
Arterial hypertension	72 (14.9)	75 (11.9)	84 (30.1)	99 (18.0)	21 (7.6)	46 (9.7)	98 (20.0)	495 (15.6)
Obesity	43 (8.9)	17 (2.7)	49 (17.6)	48 (8.7)	16 (5.8)	27 (5.7)	65 (13.3)	265 (8.3)
Cardiovascular diseases	13 (2.87)	11 (1.7)	39 (14.0)	13 (2.4)	4 (1.4)	9 (1.9)	21 (4.3)	110 (3.5)
Other STI’s	27 (5.6)	121 (19.2)	28 (10.0)	72 (13.1)	28 (10.1)	208 (44.0)	70 (14.3)	554 (17.4)

STI, sexually transmitted infections.

In terms of health status, a notable proportion of participants reported various infections and comorbidities. In addition to 955 (30%) participants previously diagnosed with HIV infection, 32 (1%) and 65 (2%) participants reported hepatitis C virus (HCV) and hepatitis B virus (HBV) infections, respectively (Table 1). Additionally, 554 (17.4%) participants reported a prior history of sexually transmitted infections. Furthermore, 268 (8.4%) participants reported a diagnosis of diabetes mellitus, while 110 (3.5%) and 265 (8.3%) reported cardiovascular diseases and obesity, respectively. Arterial hypertension was also reported by 495 (15.6%) participants (Table 1). Finally, 2.7% of participants reported a history of blood transfusion, while 0.4% reported intravenous drug use.

### Prevalence and distribution of HTLV-1 infection

Among 3,184 participants, 28 tested positive for HTLV infection based on serological analyses. These cases were confirmed by Western blotting, with 15 being identified as HTLV-1, 2 indeterminate, and 11 negative. There were two subjects who were positive for HTLV-2 in Belem. The overall prevalence of HTLV-1 was estimated to be 0.5% (95% CI: 0.3–0.8) (Table 2). The baseline characteristics of participants who were HTLV-1 positive are comprehensively described in the Supplementary Table.

**Table 2:** Prevalence of HTLV-1 by city and study population.

	City							
	Salvador *n* = 483	Belem *n* = 632	Curitiba *n* = 279	Dourados *n* = 549	Fortaleza *n* = 278	Natal *n* = 473	Vitoria *n* = 490	Total (%; 95% CI) *n* = 3,184
General population, *n* (%)	0 (0.0)	2 (1.1)	0 (0.0)	0 (0.0)	0 (0.0)	0 (0.0)	3 (1.7)	5 (0.5; 0.2–1.3)
HIV, *n* (%)	0 (0.0)	1 (0.6)	0 (0.0)	1 (0.9)	1 (0.4)	1 (0.9)	2 (1.8)	6 (0.7; 0.3–1.4)
Tuberculosis, *n* (%)	2 (2.1)	1 (1.1)	0 (0.0)	0 (0.0)	0 (0.0)	0 (0.0)	0 (0.0)	3 (0.9; 0.3–2.7)
Pregnant women, *n* (%)	0 (0.0)	0 (0.0)	0 (0.0)	1 (0.5)	0 (0.0)	0 (0.0)	0 (0.0)	1 (0.1; 0.0–0.8)
PREP, *n* (%)	0 (0.0)	0 (0.0)	0 (0.0)	0 (0.0)	0 (0.0)	0 (0.0)	0 (0.0)	0 (0.0; 0.0–1.3)
Total (%; 95% CI)	2 (0.4; 0.1–1.5)	4 (0.6; 0.2–1.6)	0 (0.0–1.4)	2 (0.4; 0.1–1.3)	1 (0.4; 0.0–2.0)	1 (0.2; 0.0–1.2)	5 (1.0; 0.4–2.4)	15 (0.5; 0.3–0.8)

**Figure 2:**
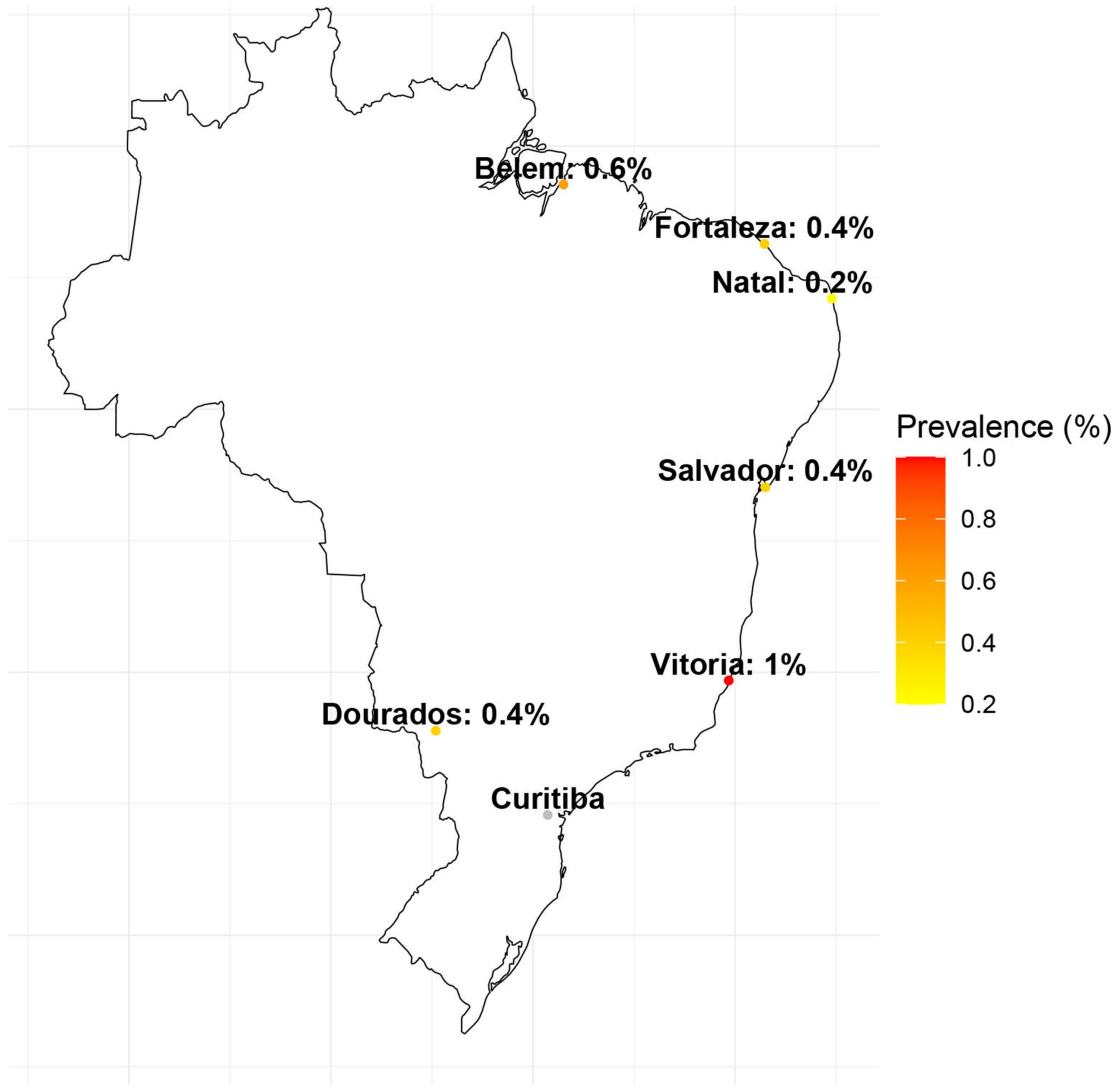
A map of Brazil showing the geographical location of the cities included in the study and their respective HTLV-1 prevalence. There was no case of HTLV-1 in the city of Curitiba.

**Figure 3:**
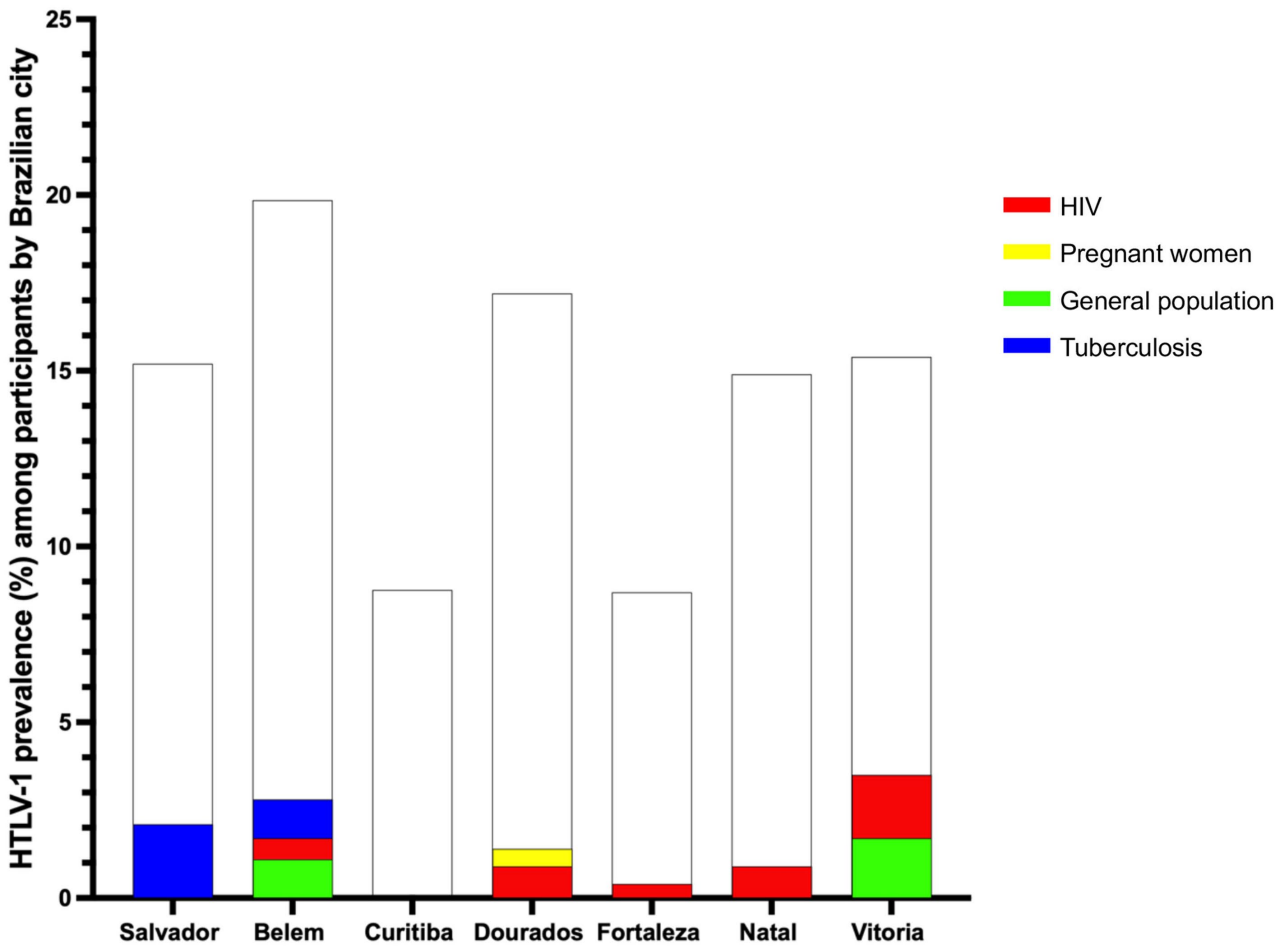
HTLV-1 prevalence among the study population, relative to the total sample size, by Brazilian city. The white bars represent the total sample size for each city relative to the overall study population. The colored bars indicate the HTLV-1 prevalence of subgroups within each city’s study population. Each color in the legend corresponds to a specific subgroup of the study population, with higher bars indicating a higher prevalence of HTLV-1 infection.

The prevalence of HTLV-1 infection in Salvador was estimated to be 0.4%, with all cases occurring among participants receiving treatment for tuberculosis, where the prevalence rate was notably higher at 2.1%. In Fortaleza and Natal, the prevalence rates were 0.4 and 0.2%, respectively, observed exclusively within the HIV study population, with a prevalence of 0.4% in Fortaleza and 0.9% in Natal. HTLV-1 prevalence in Belem was estimated to be 0.6%, distributed among the GP (1.1%), HIV-positive individuals (0.6%), and those being treated for tuberculosis (1.1%). In Dourados, the prevalence was 0.4%, identified within the HIV-positive (0.9%) and PW (0.5%) study populations. In Vitoria, the estimated prevalence of 0.6% was among the GP (1.7%) and HIV-positive individuals (1.8%). There were no HTLV-1 cases detected in the city of Curitiba (Table 2; Figures 2, 3).

Regarding the study population, the highest prevalence was observed among those receiving treatment for tuberculosis (0.9%), followed by HIV-positive individuals (0.7%), and the GP (0.5%). The prevalence among PW was 0.1% with no HTLV-1 case detected among those on PrEP (Table 2).

### Risk factors for HTLV-1 infection

**Figure 4:**
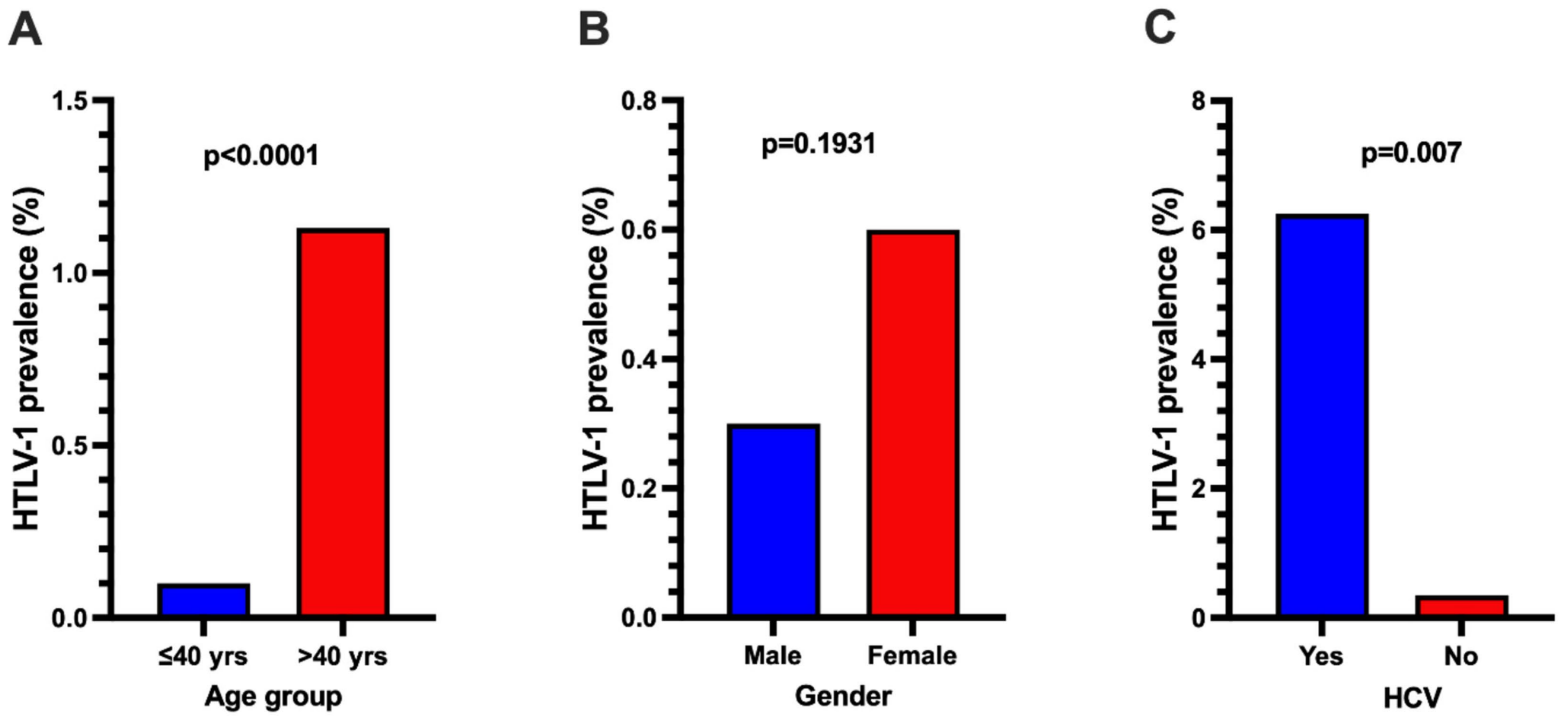
Comparison of HTLV-1 prevalence between participants with different age group **(A)**, gender **(B)**, and **(C)** those with or without prior history of HCV infection. Differences between the groups were assessed using Fisher’s Exact test, and *p* < 0.05 was considered statistically significant.

Risk factors for HTLV-1 infection were identified using univariate and multivariate logistic regression analyses. In the univariate analysis, participants over 40 years of age (OR, 11.789; 95% CI: 2.656–52.335) and those with a prior history of HCV infection (OR, 18.856; 95% CI: 3.808–93.881) were associated with significantly increased odds of HTLV-1 infection (Table 3). The prevalence rate of HTLV-1 infection among participants over 40 was significantly higher compared to those 40 years or younger (Figure 4A). Similarly, the prevalence rate of HTLV-1 infection among participants with a history of HCV infection was significantly higher compared to those without HCV infection (Figure 4C).

**Table 3:** Predictors of HTLV-1 infection among the study participants.

	Univariate logistic regression	
Predictor	Odd Ratio (95% CI)	*p* value
Age groups		
<40 years	Reference	
>40 years	11.789 (2.656–52.335)	0.001
Gender		
Male	Reference	
Female	2.394 (0.761–7.535)	0.136
Stable relationship	0.515 (0.164–1.620)	0.256
Marital status		
Single	Reference	0.197
Married	1.154 (0.325–4.099)	
Divorced/Separated	4.400 (1.092–17.724)	
Widowed	–	
Ethnicity		
Black/Mixed	Reference	0.801
White	0.762 (0.197–2.952)	
Indigenous	–	
Other	1.690 (0.435–6.564)	
Sexual orientation		
Heterosexual	1.516 (0.423–5.440)	0.523
Homosexual	0.714 (0.213–2.391)	0.585
Bisexual	1.472 (0.190–11.392)	0.711
Length of education		
≥12 years	Reference	
<12 years	–	0.992
Family income		
Up to 1 MW	Reference	0.331
2 to 5 MW	0.419 (0.133–1.318)	
More than 5 MW	–	
Comorbidities		
HCV	18.856 (3.808–93.381)	<0.001
HBV	3.609 (0.462–28.173)	0.221
Diabetes mellitus	2.108 (0.459–9.669)	0.337
Arterial hypertension	1.963 (0.519–7.427)	0.32
Obesity	–	0.995
Cardiovascular diseases	–	0.997
Other STI	1.217 (0.269–5.505)	0.799
Blood transfusion	–	0.996
Intravenous drug use	–	0.999
City		
Salvador	Reference	0.737
Belem	1.532 (0.279–8.398)	
Curitiba	–	
Dourados	0.879 (0.123–6.266)	
Fortaleza	0.868 (0.078–9.619)	
Natal	0.510 (0.046–5.638)	
Vitoria	2.479 (0.479–12.841)	
Study population		
General population	Reference	0.592
HIV	1.207 (0.367–3.967)	
Tuberculosis	1.724 (0.410–7.253)	
Pregnant women	0.258 (0.030–2.210)	
PREP	–	

In the multivariate logistic regression model, participants over 40 years of age (OR, 8.867; 95% CI: 1.824–43.099), female gender (OR, 4.604; 95% CI: 1.184–17.903), and those with a prior history of HCV infection (OR, 13.995; 95% CI: 2.374–82.506) were identified as independent risk factors of HTLV-1 infection (Table 4). Additionally, the prevalence of HTLV-1 tended to be higher in females than in males (Figure 4B).

**Table 4:** Predictors of HTLV-1 infection among the study participants.

		Multivariate	
Predictor	HTLV-1 prevalence (%)	Odd Ratio (95% CI)	*p* value
Age groups			
<40 years	0.1	Reference	
>40 years	1.1	8.867 (1.824–43.099)	0.007
Gender			
Male	0.3	Reference	
Female	0.6	4.604 (1.184–17.903)	0.028
Stable relationship	0.4	0.539 (0.146–1.987)	0.352
Marital status			
Single	0.3	Reference	0.792
Married	0.4	0.823 (0.206–3.292)	
Divorced/Separated	1.4	1.491 (0.346–6.431)	
Widowed	0	–	
Comorbidities			
HCV	6.3	13.995 (2.374–82.506)	0.004
HBV	1.5	1.698 (0.189–15.265)	0.637

## Discussion

In this study, we observed an overall prevalence rate of HTLV-1 infection of 0.5% within the entire study population. Notably, analysis across different cities revealed varying HTLV-1 prevalence rates, with the highest prevalence rate observed in Vitória (1%). HTLV-1 prevalence was most frequent among individuals with tuberculosis and HIV. Despite the lower overall HTLV-1 prevalence observed in our study compared to other previously published literature, we noted higher rates in specific groups: 1.1% among the general population in Belem, 2.1% among tuberculosis patients in Salvador, and 1.7 and 1.8% among the general population and HIV patients in Vitoria, respectively. These findings are consistent with other studies conducted in Brazil (13, 24, 25).

Further investigation identified older age (>40 years), female gender, and history of HCV infection as significant risk factors for HTLV-1 infections, underscoring the importance of socio-demographic factors and co-infections in understanding HTLV-1 epidemiology. Most importantly, the identification of older age and female gender, coupled with the high prevalence of HTLV-1 among individuals with HIV, and none among injection drug users or individuals with prior history of blood transfusions, suggests sexual transmission may be the primary route of HTLV-1 infection. In fact, studies conducted elsewhere have demonstrated a clear link between sexual intercourse and HTLV-1 transmission, corroborating our findings (26, 27).

The first study of HTLV-1 seroprevalence in Brazil, conducted among Japanese migrants in Campo Grande, revealed high endemicity (28). Since then, numerous studies have investigated HTLV-1 prevalence and distribution, often focusing on specific populations or geographical areas. Among multicenter studies, prevalence rates have varied, reflecting differences in study populations, methodologies, and geographical locations. Our study uniquely surveyed diverse populations across multiple Brazilian cities, providing a more comprehensive understanding of HTLV-1 epidemiology in the country.

Of the few multicenter studies conducted, the HTLV-1 prevalence in Salvador was 2.48 and 1.76% among hemodialysis patients and the general population (29, 30), respectively, all higher than the rate observed in our study. Similarly, a study in four cities of the state of Para reported a HTLV-1 prevalence rate of 1.7% among female sex workers (13). Conversely, a study in Mato Grosso do Sul state reported a prevalence rate of 0.2% among prisoners (31). Moreover, studies conducted in other countries such as Gabon reported a HTLV-1 prevalence rate of 8.7% among rural inhabitants within 6 provinces (32). A nationwide survey in China reported a prevalence rate of 0.005% among blood donors (33). The discrepancy in the prevalence rates between these studies and ours may be attributed to differences in the target population, study design, methodological approach, and geographical location. Our study surveyed people from diverse populations in different Brazilian cities, while these studies surveyed individuals from different centers within the same city or different countries altogether.

Interestingly, the prevalence observed in individual cities differs from those of previous studies. Salvador is recognized as the city with the highest HTLV-1 endemicity, with a recent study among the general population reporting a prevalence rate of 1.48% (24), higher than observed in our study where HTLV-1 was only prevalent among individuals with tuberculosis. Similarly, studies in Belem (34), Dourados (35), Fortaleza (36), and Vitória (25) revealed varying prevalence rates, all differing from our observed rates. Notably, our study is the first to report HTLV-1 prevalence data in the city of Natal. While previous studies focused on specific populations or high-risk groups, our study surveyed individuals from five different populations, including high-risk groups, contributing to a broader understanding of HTLV-1 epidemiology in Brazil.

To capture the true prevalence rate of HTLV-1 infection in Brazil, our study was uniquely designed to include both the high-risk and the general populations. It is worth nothing that the prevalence rate in the general population differs from similar studies in Brazil. Notably, studies conducted in Salvador (24), and Belem (34) among the general population reported prevalence rates of 1.48 and 1.4%, respectively, all of which are higher than the observed rate in this study. The larger sample sizes in these studies may account for the higher HTLV-1 prevalence. However, findings were consistent with previous studies from Vitória, which reported a prevalence of 0.5%. Furthermore, our observed prevalence rate among HIV patients was higher compared to similar studies in Midwestern Brazil (37), but lower than the prevalence rate reported in São Paulo and Pernambuco (21, 38). Moreover, a recent meta-analysis of HTLV-1 prevalence among pregnant women in Brazil revealed a rate of 0.32% (39), which is higher than the observed rate in this study. Additionally, the prevalence of HTLV-1 among tuberculosis patients was comparable to similar studies conducted in Central-West Brazil (22), highlighting the dual role of co-infections in HTLV-1 transmission. Notably, the observed prevalence differed between Salvador (2.1%) and Belem (1.1%), potentially exacerbating the risk of TB in these regions due to immune dysregulation caused by HTLV-1.

Using logistic regression analysis, we identified older age as a significant risk factor for HTLV-1 infection, a finding consistent with previous studies conducted in Brazil (40–42) and elsewhere (32). In general, age serves as a crucial risk factor for contracting infectious pathogens due to longer cumulative exposure time. In the case of HTLV-1, transmission primarily occurs through sexual intercourse, blood transfusion/sharing needles, and breastfeeding. Hence, longer exposure to these routes over time can increase the likelihood of infection, especially in regions with high endemicity.

Our study showed that female gender is associated with an increased risk of contracting HTLV-1 infection, corroborating similar study in Brazil (43) and elsewhere (32). The virus replicates at a significantly higher rate in seminal fluid, resulting in higher levels of infected lymphocytes in semen than in vaginal fluids (44, 45). This disparity facilitates more efficient transmission of the virus from men to women than vice versa, thus making women more highly susceptible to contracting the virus though long-term exposure through the sexual route.

HCV infection was also identified as a significant risk factor for HTLV-1 infection, and this is consistent with a similar study in Brazil (46). Our data suggests a potential interaction between these two viruses. Although the underlying mechanism is not known, the association between HCV and HTLV-1 may be attributed to similar routes of transmission, including intravenous drug use, contaminated blood or blood products, and sexual contact. Additionally, HCV infection can lead to chronic liver disease, which may compromise the immune system and thus increase susceptibility to other infections, including HTLV-1. Moreover, HCV-infected individuals may be more likely to undergo regular medical monitoring and testing, leading to increased detection of co-existing HTLV-1 infection through routine screening. Conversely, we have shown that HTLV-1 infection increases the likelihood of HCV spontaneous clearance (47, 48). Future studies that focus on immune responses of HTLV-1 HCV co-infected subjects will help elucidate the interactions between these two viruses (49).

This study suggests that the primary mode of HTLV-1 transmission is likely through the sexual route, consistent with findings from studies in Brazil (24) and other countries (50, 51). This hypothesis is supported by the absence of HTLV-1 among participants with a history of blood transfusion or intravenous drug use, thus ruling out the parenteral route. Additionally, the identification of older age and female gender as significant predictors of HTLV-1 infection by logistic regression modeling rules out vertical transmission of the virus. In fact, about 87% of the HTLV-1 cases were among participants over 40 years, and 73% occurred in females. The prevalence was also higher among the HIV-positive individuals.

One of the strengths of this study is the inclusion of participants from different cities and study populations, as it provides a broader perspective on the prevalence and risk factors associated with HTLV-1 infection. This approach allows for a more comprehensive understanding of the epidemiology of HTLV-1 in diverse settings and populations, enhancing the generalizability of our findings. However, it is important to acknowledge several limitations of the study. First, we focused exclusively on individuals attending specialized healthcare facilities, potentially introducing selection bias, and limiting the generalizability of our findings to the general population. Thus, including individuals outside these healthcare facilities would provide a more representative sample and a more accurate estimate of HTLV-1 prevalence. It is worth noting, however, that the included population should have a higher risk of HTLV infection, according to the available evidence, and a higher prevalence, which was not detected in our study. Second, the cross-sectional design of the study limits our ability to establish causality or temporal relationships between HCV and HTLV-1 infection. Longitudinal studies are needed to elucidate the dynamics of co-infection and the impact of one virus on the acquisition or progression of the other. Furthermore, the wider confidence interval observed for HCV as a predictor of HTLV-1 infection highlights uncertainty in the effect estimates. This wider interval may be due to relatively small sample size, limiting the precision of the findings. However, HCV has been shown to be a risk factor for HTLV-1 infection in Brazil (46). Finally, the reliance on self-reported data for risk factors may introduce recall bias and underreporting, potentially affecting the accuracy of our results. Future studies could incorporate more objective measures or validation techniques and larger sample size to improve the precision and reliability of risk factor data. Despite these limitations, our study provides valuable insights into the association between HCV and HTLV-1 infection and emphasizes the need for further research to elucidate the underlying mechanisms and implications of this relationship for clinical management and public health interventions. In addition, it shows that the prevalence of HTLV-1 infection in Brazil may be lower than that previously reported and reinforces the sexual exposure as the primary route of transmission.

In conclusion, our study provides compelling evidence for the variability of HTLV-1 infections across different geographical locations, and among diverse study populations in Brazil. We identified older age, female gender, and HCV as significant risk factors for HTLV-1 infection, highlighting the critical role of socio-demographic factors and co-infections in its epidemiology. Furthermore, we identified sexual transmission as the primary route of HTLV-1 infection. These findings emphasize the need for targeted interventions and public health strategies addressing age-related risks, socio-economic disparities, co-infection dynamics, and safe sex practices to effectively control HTLV-1 transmission. By understanding the interplay of these factors, we can better tailor prevention and screening programs to mitigate the burden of HTLV-1 infections and improve overall public health outcomes.

## Data Availability

The original contributions presented in the study are included in the article/Supplementary material, further inquiries can be directed to the corresponding author.
