# Supplementary material for: Multicenter cross-sectional study of HTLV-1 prevalence and associated risk factors in epidemiologically relevant groups across Brazil
Source: Front Public Health. 2025 Mar 3;13:1511374. doi: 10.3389/fpubh.2025.1511374 (PMC11911192; doi:10.3389/fpubh.2025.1511374)
Supplement: Supplementary file 1 [file Table_1.DOCX]

Supplementary Table. Baseline characteristics of particpants with HTLV-1 infection

|  | Total  n= 3184 | HTLV-1 positive cases  n= 28 |
| --- | --- | --- |
| **Age groups, n (%)** |  |  |
| Up to 40 | 2037 (64.0) | 2 (0.1) |
| More than 40 | 1135 (35.7) | 13 (1.1) |
| **Gender, n (%)** |  |  |
| Male | 1478 (46.4) | 4 (0.3) |
| Female | 1704 (53.5) | 11 (0.6) |
| Stable relationship, **n (%)** | 1523 (47.8) | 6 (0.4) |
| **Marital Status, n (%)** |  |  |
| Single | 1802 (56.6) | 6 (0.3) |
| Married | 1040 (32.7) | 4 (0.4) |
| Divorced/Separated | 207 (6.5) | 3 (1.4) |
| Widowed | 91 (2.9) | 0 (0.0) |
| **Ethnicity, n (%)** |  |  |
| Black/Mixed | 1699 (53.4) | 7 (0.4) |
| White | 955 (30.0) | 3 (0.3) |
| Indigenous | 432 (13.6) | 0 (0.0) |
| Other | 21 (0.6) | 3 (14.3) |
| **Sexual Orientation, n (%)** |  |  |
| Heterosexual | 1949 (61.2) | 9 (0.5) |
| Homosexual | 542 (17.0) | 3 (0.6) |
| Bisexual | 155 (4.9) | 1 (0.6) |
| **Length of Education, n (%)** |  |  |
| <12 years | 651 (20.4) | 13 (2.0) |
| ≥12 years or more | 2461 (77.3) | 0 (0.0) |
| **Family Income, n (%)** |  |  |
| Up to 1 MW | 1499 (47.1) | 11 (0.7) |
| 2 to 5 MW | 1296 (40.7) | 4 (0.3) |
| More than 5 MW | 382 (12.0) | 0 (0.0) |
| **Blood Transfusion** | 85 (2.7) | 0 (0.0) |
| **Intravenous drug use** | 11 (0.4) | 0 (0.0) |
| **Comorbidities, n (%)** |  |  |
| HCV | 32 (1.0) | 2 (6.3) |
| HBV | 65 (2.0) | 1 (1.5) |
| Diabetes Mellitus | 268 (8.4) | 2 (0.7) |
| Arterial Hypertension | 495 (15.6) | 3 (0.6) |
| Obesity | 265 (8.3) | 0 (0.0) |
| Cardiovascular Diseases | 110 (3.5) | 0 (0.0) |
| Previous STI | 554 (17.4) | 1 (0.2) |
